# Supplementary material for: SIG-1451, a Novel, Non-Steroidal Anti-Inflammatory Compound, Attenuates Light-Induced Photoreceptor Degeneration by Affecting the Inflammatory Process
Source: Int J Mol Sci. 2022 Aug 8;23(15):8802. doi: 10.3390/ijms23158802 (PMC9369167; doi:10.3390/ijms23158802)
Supplement: Supplementary file 1 [file ijms-23-08802-s001.zip › ijms-1745032-supplementary.pdf]

Supplementary Figures

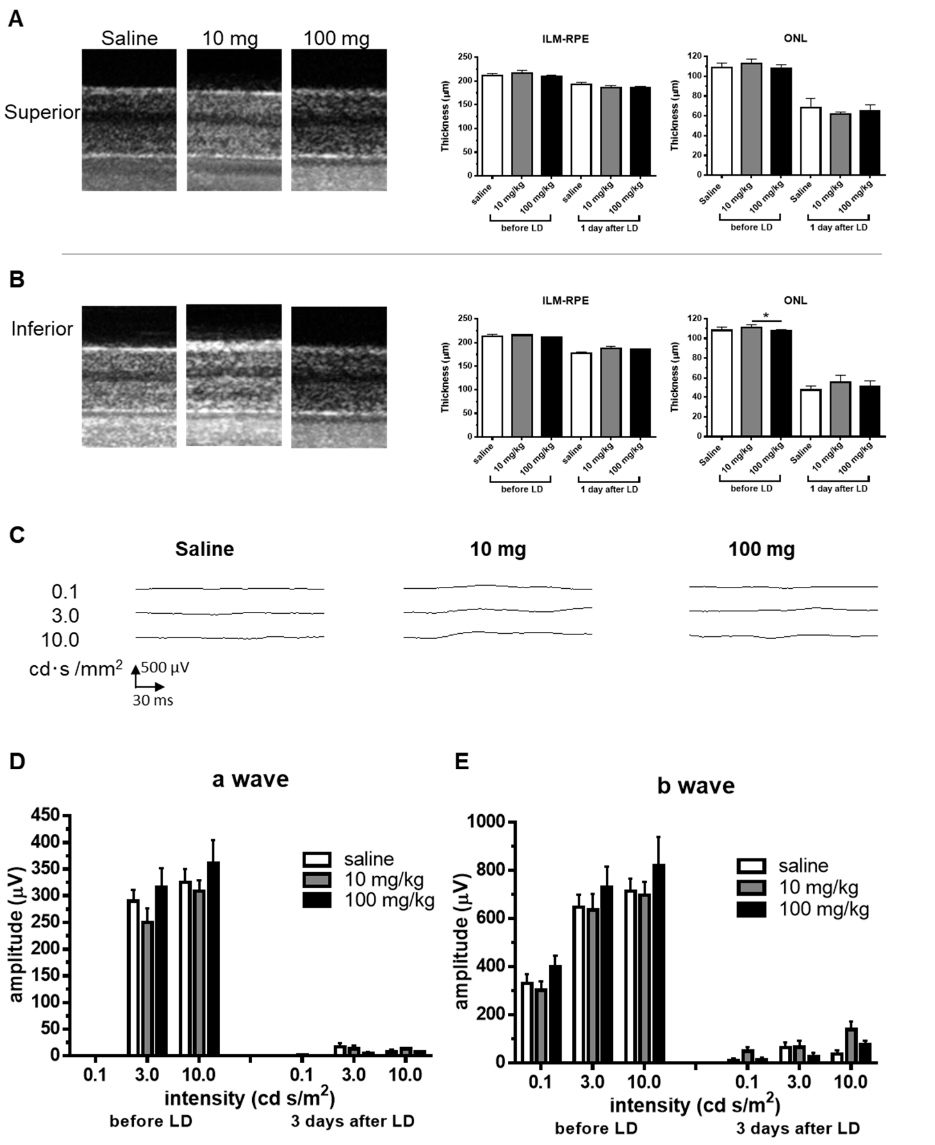

Figure S1. Effects of SIG-1452 on light-induced photoreceptor degenerations

As a preliminary experiments, 3000 lux of light for 24 hours were exposed to rats and 10 mg/kg or 100 mg/kg of SIG-1451 was intraperitoneally administered to the rats based on the experimental schedules shown in Fig. 1S. OCT images were obtained from the superior- (A) and the inferior part (B) of retinas 1 day after the end of the light exposure, The thickness of the inner limiting membrane ILM) from retinal pigment epithelium (RPE) and the outer nuclear layers including outer segments (ONL) were measured. Typical waveforms of ERGs were shown in (C). Amplitudes of a-wave (D) and b-wave (E) were measured. Data are shown as mean ± SEM (Saline: n=3, 10 mg/kg SIG-1451: n=3, 100 mg/kg SIG-1451: n=4).

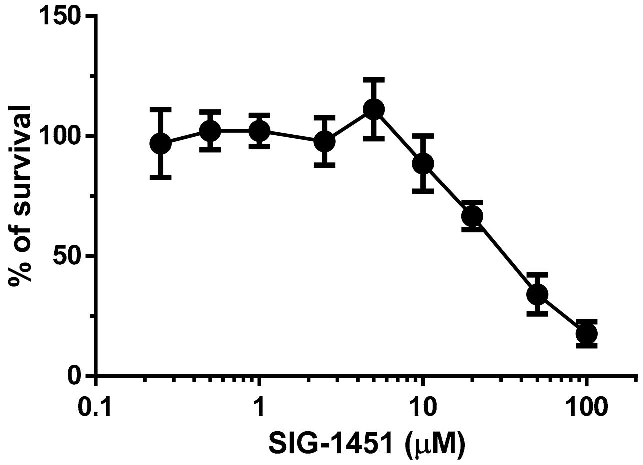

Figure S2. Toxicities of SIG-1451 on cultured Müller cells

Independent experiments were performed and all data was shown in the graph (n=10, mean ± SD).

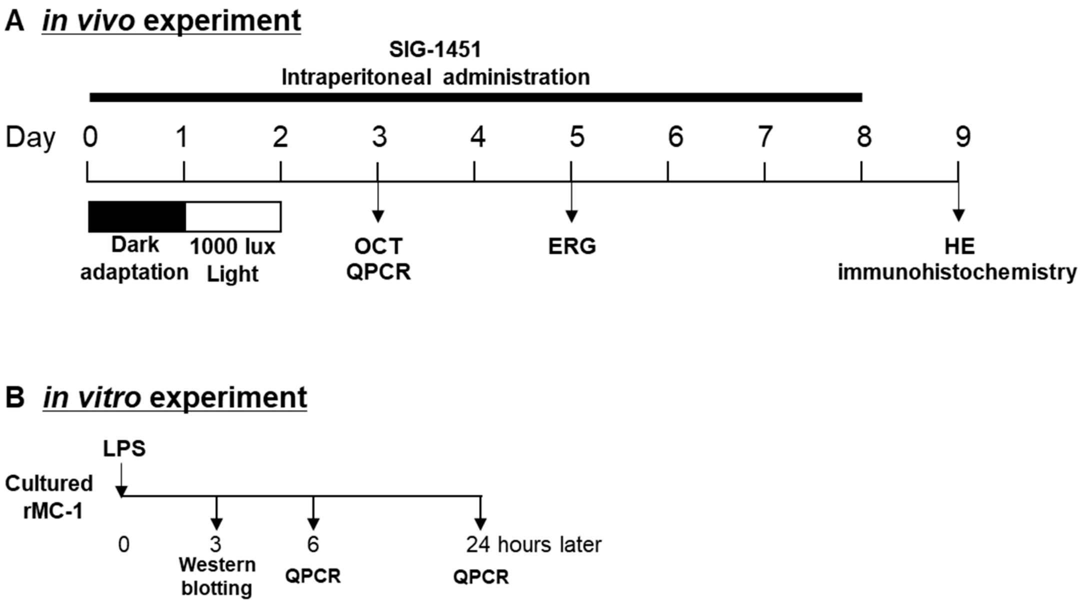

Figure S3. Experimental designs in vivo (A) and in vitro (B)
